# Shared immune-inflammatory gene networks and drug prediction in polycystic ovary syndrome and type 2 diabetes mellitus: a bioinformatics and experimental validation study

**DOI:** 10.3389/fendo.2026.1747045

**Published:** 2026-04-16

**Authors:** Xuemeng Liu, Yanlei Wang, Qiuhan Bi, Ying Zhang, Shumin Wang, Pan Yang, Jie Pei, Weixi Zhu, Yijing Chen, Zhiguo Zhang, Beili Chen, Qiu Zhang, Yi Zhang, Tian Jiang

**Affiliations:** 1 Department of Science and Education, The Second People’s Hospital of Hefei, Hefei Hospital Affiliated to Anhui Medical University, Hefei, Anhui, China; 2 Bengbu Medical University, Bengbu, Anhui, China; 3 Department of Endocrinology, The First Affiliated Hospital of Anhui Medical University, Hefei, Anhui, China; 4 Department of Obstetrics and Gynecology, The First Affiliated Hospital of Anhui Medical University, Hefei, Anhui, China

**Keywords:** drugs prediction, gene regulatory networks, immunity, inflammation, polycystic ovary syndrome, type 2 diabetes mellitus

## Abstract

**Background:**

Polycystic ovary syndrome (PCOS) is associated with an increased risk of type 2 diabetes mellitus (T2DM), and the risk of PCOS increases in patients with T2DM of reproductive age. The bidirectional link between PCOS and T2DM has been confirmed through experimental and epidemiological evidence; however, the genetic factors that contribute to deeper insights into the shared pathogenesis of these two diseases remain unclear. We aimed to identify shared immune- and inflammation-related genes and pathways in PCOS and T2DM, further explore the molecular mechanisms in developing this comorbidity, and predict drugs with potential effects to develop novel therapeutic strategies.

**Methods:**

We obtained microarray expression profiling datasets (GSE34526 and GSE25724) of PCOS and T2DM from the Gene Expression Omnibus (GEO) database. The differential expression genes (DEGs) between disease and control groups were identified and analyzed via the R package “limma” following data preprocessing. The R package “clusterProfiler” was applied to conduct Gene ontology (GO) and Kyoto Encyclopedia of Genes and Genome (KEGG) pathway enrichment analyses. Hub genes were identified from the protein-protein interaction (PPI) network using the Molecular Complex Detection (MCODE) and cytoHubba plug-ins of Cytoscape. Transcription factor (TF)-hub and miRNA-hub gene regulatory networks were constructed and visualized using Cytoscape. The Drug-Gene Interaction Database (DGIdb) was used to predict prospective drugs targeting hub genes. In addition, hub genes were verified by RT-qPCR.

**Results:**

A total of 239 common DEGs, including 140 upregulated genes and 99 downregulated genes, were discovered. These common DEGs were primarily associated with immune regulation and inflammatory processes. Moreover, ITGAM, ITGB2, SPI1, C1QB, CCR5, C3AR1, LY86, AIF1, and IRF8 were identified as hub genes and the RT-qPCR results showed significant differences. These hub genes were predominantly related to the regulation of neutrophil degranulation (ITGAM, ITGB2, and SPI1), dendritic cell chemotaxis (CCR5 and SPI1), follicular B cell differentiation (SPI1 and IRF8), synapse pruning (ITGAM and C1QB), integrin αM-β2 complex (ITGAM and ITGB2), the regulation of prostaglandin-E synthase activity (ITGAM and ITGB2), *Staphylococcus aureus* infection (ITGAM, ITGB2, C1QB, and C3AR1) and pertussis (IRF8). Finally, we predicted 19 TFs, 170 miRNAs, and 40 potential therapeutic drugs interacting with hub genes.

**Conclusion:**

We identified nine hub genes and related gene regulatory networks and discussed novel perspectives on the roles of immunity and inflammation in patients with PCOS and T2DM. Moreover, maraviroc, cenicriviroc, PF-04634817 (targeting CCR5), butein (targeting ITGB2), dimethyl sulfoxide (targeting ITGAM), and rovelizumab (targeting both ITGB2 and ITGAM) are potential therapeutic drugs. However, these findings require validation through further clinical and experimental studies.

## 1 Introduction

Polycystic ovary syndrome (PCOS) is a common reproductive and metabolic endocrine disorder with a high incidence rate of 6–20% in reproductive-aged women and characterized by chronic ovarian dysfunction (oligo/anovulation), hyperandrogenism (biochemical or clinical), and polycystic ovarian morphology (1). PCOS has high phenotypic heterogeneity associated with menstrual disorders, infertility, and long-term metabolic complications, including insulin resistance (IR), impaired glucose tolerance (IGT), type 2 diabetes mellitus (T2DM), abdominal obesity, dyslipidemia, cardiovascular diseases (CVDs) and non-alcoholic fatty liver disease (NAFLD) (2, 3). Long-term reproductive, metabolic, and psychological stress caused by PCOS imposes a substantial economic and health burden on families and society (4). In 2020, the annual economic cost of T2DM attributed to PCOS was $1,500 million in the United States (5).

T2DM is a chronic metabolic disease characterized by hyperglycemia, IR, and pancreatic β-cell dysfunction (6). In 2015, the number of patients with DM aged 20–79 years was 415 million worldwide, of which over 90% had T2DM, with an expected rise to 642 million by 2040 (7). T2DM places considerable economic pressure on both individuals and society. The global cost of DM was estimated to reach $ 673 billion in 2015, with a predicted rise to $ 802 billion by 2040 (7). T2DM is an important metabolic complication of PCOS (8). Women with PCOS have a higher incidence of T2DM and IGT compared to a control cohort matched based on body mass index (BMI) (9–11). Furthermore, animal studies have revealed elevated fasting blood glucose concentrations and IR in PCOS rat models (12, 13). T2DM is also a significant risk factor for PCOS (14). A recent meta-analysis including 470 girls indicated that approximately 20% of girls with T2DM (average age of initial diagnosis, 12.9–16.1 years) had experienced PCOS (15). This number is significantly higher than PCOS prevalence in general adolescent females, estimated at 1.14–10% (16, 17). The term “comorbidity” is gradually being used to describe the bidirectional link between PCOS and T2DM (18, 19). Therefore, investigating this bidirectional relationship between PCOS and T2DM will increase the understanding of the pathogenesis and therapy for these two diseases.

Earlier research has provided strong evidence that IR is a shared feature of PCOS and T2DM (14, 20). In women with PCOS, IR is primarily responsible for the development of IGT and T2DM (21). Generally, IR is estimated to exist in 80–100% of T2DM patients, 70% of obese PCOS women, and 30% of lean PCOS women (14, 22). Cassar et al. reported that women with PCOS have a 27% decrease in insulin sensitivity regardless of age or BMI (4). Furthermore, as a major pathogenesis of PCOS and its metabolic complications, hyperandrogenism can interact with IR and increase the risk of T2DM (1, 23). A Swedish study indicated that T2DM risk in women with the hyperandrogenic PCOS phenotype was 3–4 times that in women without the hyperandrogenic PCOS phenotype (23). This may be due to IR worsening caused by hyperandrogenism. However, the common molecular mechanisms underlying PCOS and T2DM still need further exploration.

Recently, microarray technology and bioinformatics analysis have garnered increasing attention. These technologies enable the rapid screening and analysis of tens of thousands of gene expression data across various diseases, which helps to explore the common pathogenesis between diseases more deeply at the genetic level. Common immune- and inflammation-related hub genes that are differentially expressed in PCOS and T2DM were discovered in this study. In addition, transcription factor (TF)-hub and miRNA-hub gene regulatory networks were constructed to identify shared molecular signatures and underlying mechanisms associated with PCOS and T2DM. Finally, we proposed potential therapeutic drugs. This study is the first to offer new insights regarding shared molecular mechanisms and therapeutic strategies for patients with PCOS and T2DM from the perspectives of immunity and inflammation, which is of clinical value.

## 2 Materials and methods

### 2.1 Microarray data acquisition

**Figure 1:**
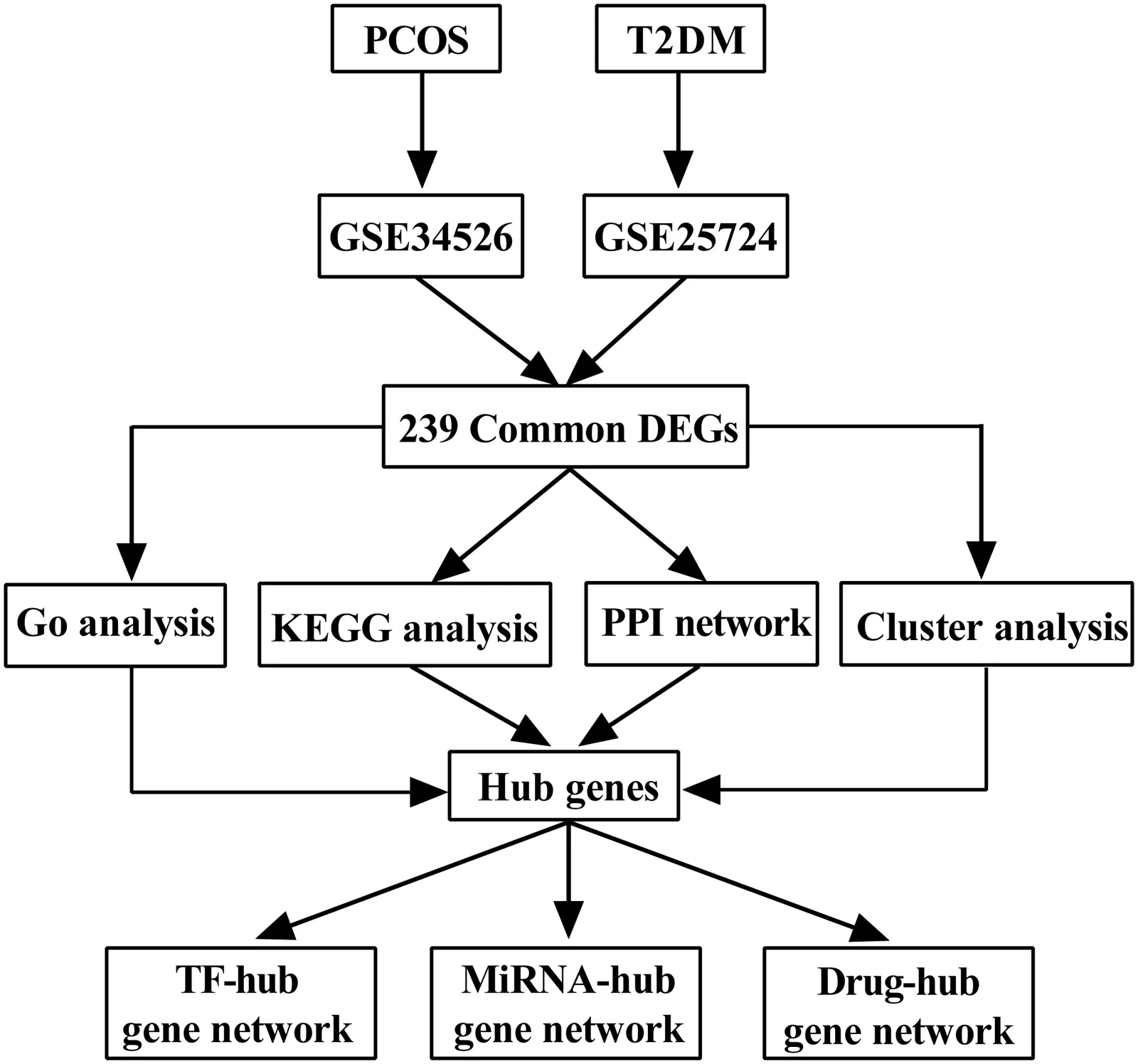
Flow chart of this work.

Based on the GPL570 and GPL96 platforms, we retrieved two microarray expression profiling datasets (GSE34526 and GSE25724) pertaining to PCOS and T2DM from the Gene Expression Omnibus (GEO) public database (https://www.ncbi.nlm.nih.gov/gds). All the keywords listed in **Table 1** were used to select the datasets. The GSE34526 dataset, obtained from follicular granulosa cells, contains ten independent samples of seven patients with PCOS and three normal controls. The GSE25724 dataset, obtained from pancreatic islet β cells, contains six independent samples of three patients with T2DM and three normal controls. Annotation files for GPL570 and GPL96 were also obtained from the GEO database. During the data collection process, only female participants were included, considering the sex-based effects of PCOS. A flowchart of this work is illustrated in **Figure 1**, and the characteristics of the two datasets are presented in **Table 2**.

**Table 1:** Electric search strategy in GEO.

Database	GEO	GEO
Disease	PCOS	Type 2 DM
Search Term	[“polycystic ovary syndrome” (MeSH Terms)] AND [“granulosa cells” (MeSH Terms)] AND [“Homosapiens” (porgn: txid9606)] AND [“Expression profiling by array” (Filter)]	[“type 2 diabetes” (All Fields)] AND [“islets” (All Fields)] AND [“tissues” (All Fields)] AND [“female” (All Fields)]AND [“Homo-sapiens” (porgn: txid9606)] AND [“Expression profiling by array” (Filter)]
Filter	Expression profiling by array Homosapiens	Expression profiling by array Homosapiens
Results	7	6

**Table 2:** Characteristics of selected datasets.

GSE ID	GSE34526		GSE25724	
Group	Normal	PCOS	Normal	Type 2 DM
Type of cells (homo sapiens)	Follicular granulosa cells		Pancreatic islet β cells	
Platform	GPL570 Affymetrix Human Genome U133 Plus 2.0 Array		GPL96 Affymetrix Human Genome U133A Array	
Number of samples	3	7	3	3
Gender	female		female	
Age (mean±SD) (year)	27.50 ± 2.36	29.29 ± 2.96	75.33±2.08	67.33±11.59
BMI (mean±SD) (kg/m^2^)	22.90 ± 1.06	25.76±4.90	23.90±1.95	26.47±2.55
References	Kaur S et al.		Dominguez V et al.	

### 2.2 Data preprocessing

First, the raw data were preprocessed using the robust multi-array average (RMA) algorithm of the “Affy” package in the R language. RMA comprises background correction, quantile normalization, and expression calculations (24). The probe ID was annotated as a gene symbol based on the platform annotation files. Probes that did not match specific gene symbols were removed, and the average level of the probes was adopted as the gene expression value when the same gene symbol corresponded to multiple probes. Batch effects in the datasets were removed using the “sva” package (25).

### 2.3 Differential expression analysis

After preprocessing the data, the differential expression genes (DEGs) between disease and control groups of each dataset were identified and analyzed via the “limma” R package (26). Significant DEGs were considered based on |log_2_ fold change (FC) | > 0.5 and an adjusted *p*-value < 0.05. A Venn diagram of the overlapping DEGs between the PCOS and T2DM groups was created using the Venn online tool (http://www.ehbio.com/test/venn/) (27). The DEGs with log_2_ FC > 0.5 were regarded as upregulated genes, while those with log_2_ FC < -0.5 were regarded as downregulated. The heat maps and volcano plots of the DEGs were depicted using the R packages “pheatmap” and “ggplot2”, respectively (28).

### 2.4 Functional and pathway enrichment analyses of common DEGs

Gene Ontology (GO) and Kyoto Encyclopedia of Genes and Genome (KEGG) pathway enrichment analyses were conducted using the “clusterProfiler” package (29). GO analysis comprised three categories based on gene function: biological processes (BP), cellular components (CC), and molecular functions (MF) (30). KEGG pathway analysis can help to construct molecular interactions, reactions, and relationship networks (31). The common DEGs were included in GO and KEGG pathway enrichment analyses, which were conducted for the up and downregulated DEGs, respectively. Terms with ≥ 2 enriched DEGs and an adjusted *p*-value < 0.05 were selected. The *P*-values were adjusted using Benjamini–Hochberg method.

### 2.5 Protein-protein interaction network construction and modules selection

The PPI network was constructed to further explore interactions among common DEGs using the Search Tool for the Retrieval of Interacting Genes (STRING, version 11.5.0, http://string-db.org) database (32). PPI pairs with an interaction score > 0.4 were selected, and Cytoscape software was employed to visualize the PPI network (33). The NetworkAnalyzer plug-in of Cytoscape was used to calculate degree scores for each node (34). Subsequently, the Molecular Complex Detection (MCODE) plug-in of Cytoscape was employed to identify key modules of the PPI network. The filter criteria were as follows: degree cutoff = 2, node score cutoff = 0.2, k-core = 2, and max depth = 100 (35).

### 2.6 Identification and analysis of hub genes

Hub genes were screened using the cytoHubba plug-in of Cytoscape (36). Based on the Maximal Clique Centrality (MCC) algorithm and degree score, the top 10 hub genes were selected separately. The related GO and KEGG pathway enrichment analyses of hub genes were visualized using the GlueGO plug-in of Cytoscape (37).

### 2.7 Construction of miRNA-hub gene network and transcription factor-hub gene network

The upstream miRNAs of the hub genes were predicted using the following four databases: miRDB (https://mirdb.org/) (38), targetscan 8.0 (https://www.targetscan.org/mamm_31/) (39), mirDIP (https://ophid.utoronto.ca/mirDIP/) (40), and miRWalk 3.0 (http://mirwalk.umm.uni-heidelberg.de/) (41). To improve the accuracy of the predicted miRNAs, the following filter criteria were set: miRDB (target prediction score > 80), targetscan 8.0 (context ++ score ≤ −0.2), mirDIP (score class: high, integrated score > 0.3), miRWalk 3.0 (the score ≥ 0.95). Subsequently, the miRNAs matching two or more databases were selected. The iRegulon plug-in of Cytoscape was employed to predict TFs regulating hub genes (42). The cutoff criteria were as follows: enrichment score threshold = 3.0, ROC threshold for AUC calculation = 0.03, rank threshold = 5,000, minimum identity between orthologous genes = 0.05, maximum false discovery rate on motif similarity = 0.001, and normalized enrichment score > 4.5. Finally, miRNA-hub and TF-hub gene regulatory networks were constructed using Cytoscape software (33).

### 2.8 Identification of potential therapeutic drugs for hub genes

The Drug-Gene Interaction Database (DGIdb, version 4.2.0, https://www.dgidb.org/) is a free resource that provides a platform for predicting drug-gene interactions and druggable gene categories from databases, publications, and other web-based sources (43). In this study, potential therapeutic drugs targeting hub genes were screened through DGIdb. The strength of the drug-gene interactions was evaluated based on interaction scores. Afterward, the drug-hub gene interaction network was visualized by Cytoscape software (33).

### 2.9 Experimental validation of hub genes in clinical samples

To perform experimental validation of hub genes, we recruited women undergoing *in vitro* fertilization (IVF) treatment, including 14 healthy controls, 11 patients with PCOS and 3 patients with PCOS and diabetes. The 14 healthy controls were women without PCOS and T2DM, and they were undergoing IVF due to male factor infertility. All participants recruited in this study were of Chinese Han ethnicity, and their ages ranged from 25 to 38 years. The diagnostic criteria for PCOS were proposed in Rotterdam in 2003. PCOS can be diagnosed when two or three of the following features exist: oligo- and/or anovulation, clinical and/or biochemical hyperandrogenism, and polycystic ovarian morphology (44). According to the 2021 American Diabetes Association (ADA) guidelines (45), the diagnostic criteria for T2DM are as follows: fasting plasma glucose ≥7.0 mmol/L or random plasma glucose ≥ 11.1 mmol/L or 2-hour plasma glucose ≥ 11.1 mmol/L during oral glucose tolerance test (OGTT) or glycosylated hemoglobin A1C ≥ 6.5%. Exclusion criteria included individuals with other reproductive endocrine disorders, such as hyperthyroidism, hypothyroidism, adrenocortical hyperfunction/insufficiency, hyperprolactinemia. Patients with congenital adrenal hyperplasia, hypogonadotropic hypogonadism, gonadal dysplasia, endometriosis, uterine fibroids, ovarian insufficiency, ovarian tumors, and luteal insufficiency were also excluded. All patients visited the First Affiliated Hospital of Anhui Medical University. Follicular granulosa cells were isolated from follicular fluid by Ficoll density centrifugation. This study was approved by Ethics Committee of the First Affiliated Hospital of Anhui Medical University.

### 2.10 Quantitative real-time polymerase chain reaction and statistical analysis

Total RNA was extracted from isolated follicular granulosa cells using Trizol reagent (Biosharp, Anhui). Reverse transcription was performed with ToloScript All-in-one RT EasyMix (Tolobio, Shanghai) on a A300 Fast Gradient Thermal Cycler system (LongGene, Hangzhou), followed by PCR amplification using 2×Q3 SYBR qPCR Master Mix (Tolobio, Shanghai) on a ThermoFisher Applied Biosystems 7500 Real-Time PCR system (ThermoFisher, USA). 18S rRNA was employed as the normalization reference gene. The relative mRNA expression levels of target genes were calculated using the 2-ΔΔCT method. The primer sequences of all genes used in this study are detailed in **Table 3**. Statistical analysis of RT-qPCR data was performed using ANOVA in GraphPad Prism, with a p-value < 0.05 considered statistically significant.

**Table 3:** The primer sequences used in this study.

Gene	Forward primer sequence (5′-3′)	Reverse primer sequence (5′-3′)
18SRNA	CTCAACACGGGAAACCTCAC	AGACAAATCGCTCCACCAAC
ITGAM	GCCTTGACCTTATGTCATGGG	CCTGTGCTGTAGTCGCACT
ITGB2	GGACCTCTCCTACTCCATGC	GCTGGCACTCTTTCTCCTTG
SPI1	GCGACCATTACTGGGACTTCC	GGGTATCGAGGACGTGCAT
C1QB	ATGGGGCAGCATCCCAGTA	CTCCCTTCTCTCCGAACTCAC
CCR5	GTTGGACCAAGCTATGCAGGT	GCAGAAGCGTTTGGCAATGT
C3AR1	CCCTACGGCAGGTTCCTATG	GACAGCGATCCAGGCTAATGG
LY86	TCACAGCCACTCTCTTCCTCT	GTAATGGATCGCAACTCTGGTAG
AIF1	ATGAGCCAAACCAGGGATTTAC	GGGATCGTCTAGGAATTGCTTGT
IRF8	AGTAGCATGTATCCAGGACTGAT	CACAGCGTAACCTCGTCTTC

## 3 Results

### 3.1 Identification of common DEGs in PCOS and T2DM

**Figure 2:**
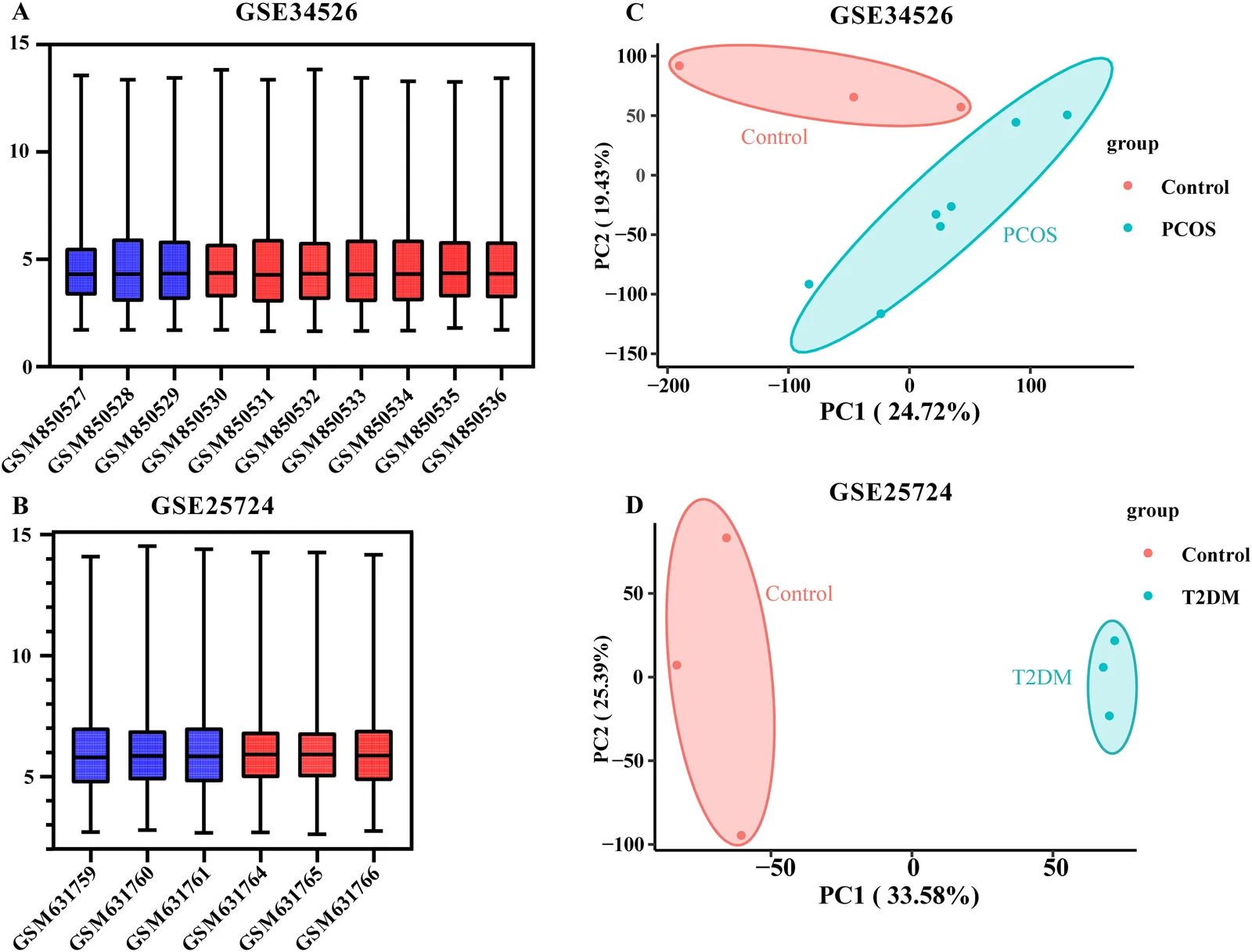
Boxplots and principal component analysis (PCA) of dataset samples after background adjustment and normalization. **(A, B)** Boxplots of GSE34526 and GSE25724 datasets: the data distributions were neat. The disease group is marked in red, and the control group is marked in blue; **(C, D)** PCA of two datasets: obvious stratification was observed between the disease and control groups.

**Figure 3:**
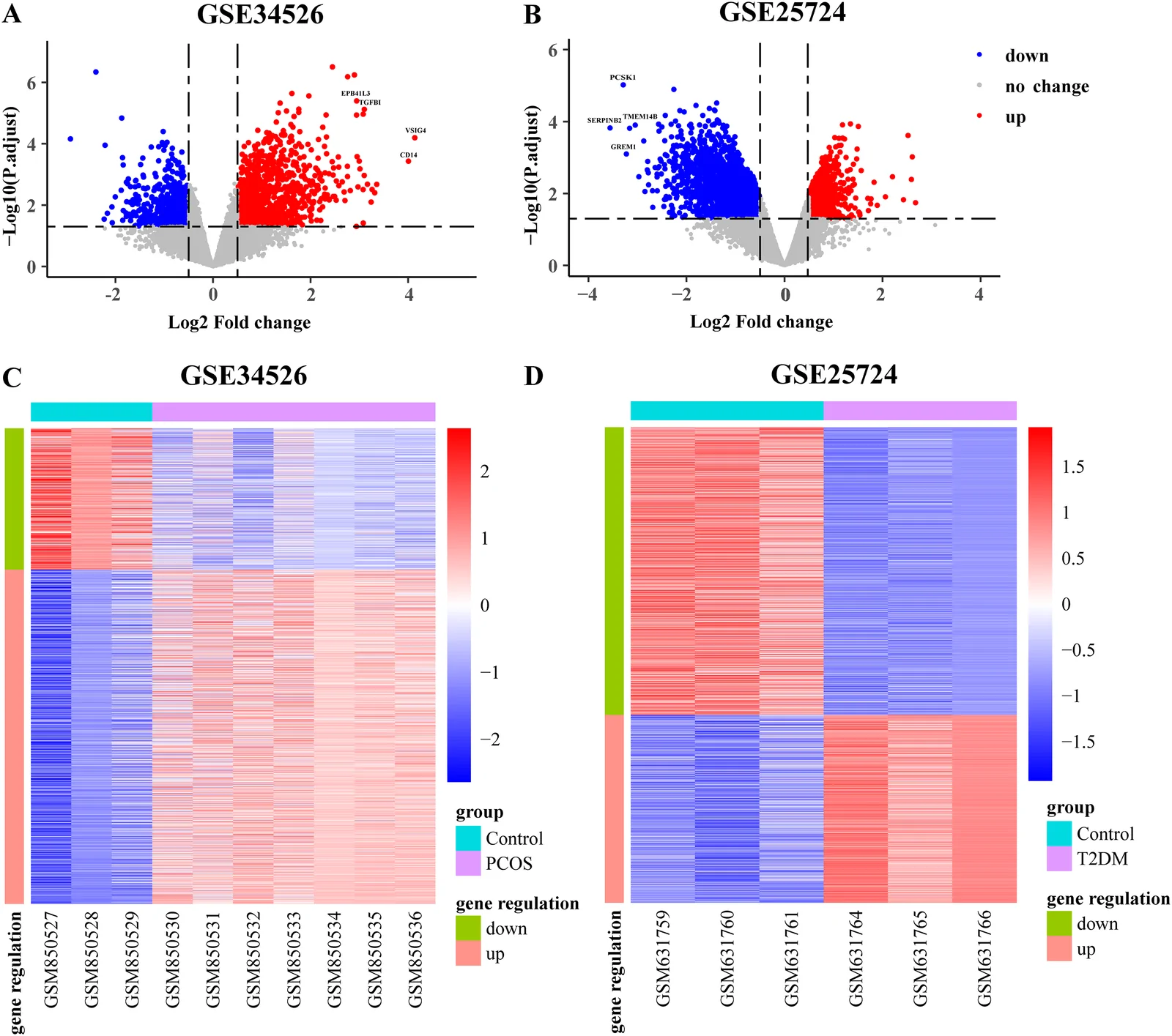
Volcano plots and heatmaps of differentially expressed genes (DEGs). **(A, B)** The volcano plots of DEGs in each dataset, |log_2_ fold change (FC) | > 0.5, adjusted *p*-value < 0.05; **(C, D)** The heatmaps of DEGs in each dataset. Red represents upregulated DEGs, blue represents downregulated DEGs, and gray represents genes with no significance.

**Figure 4:**
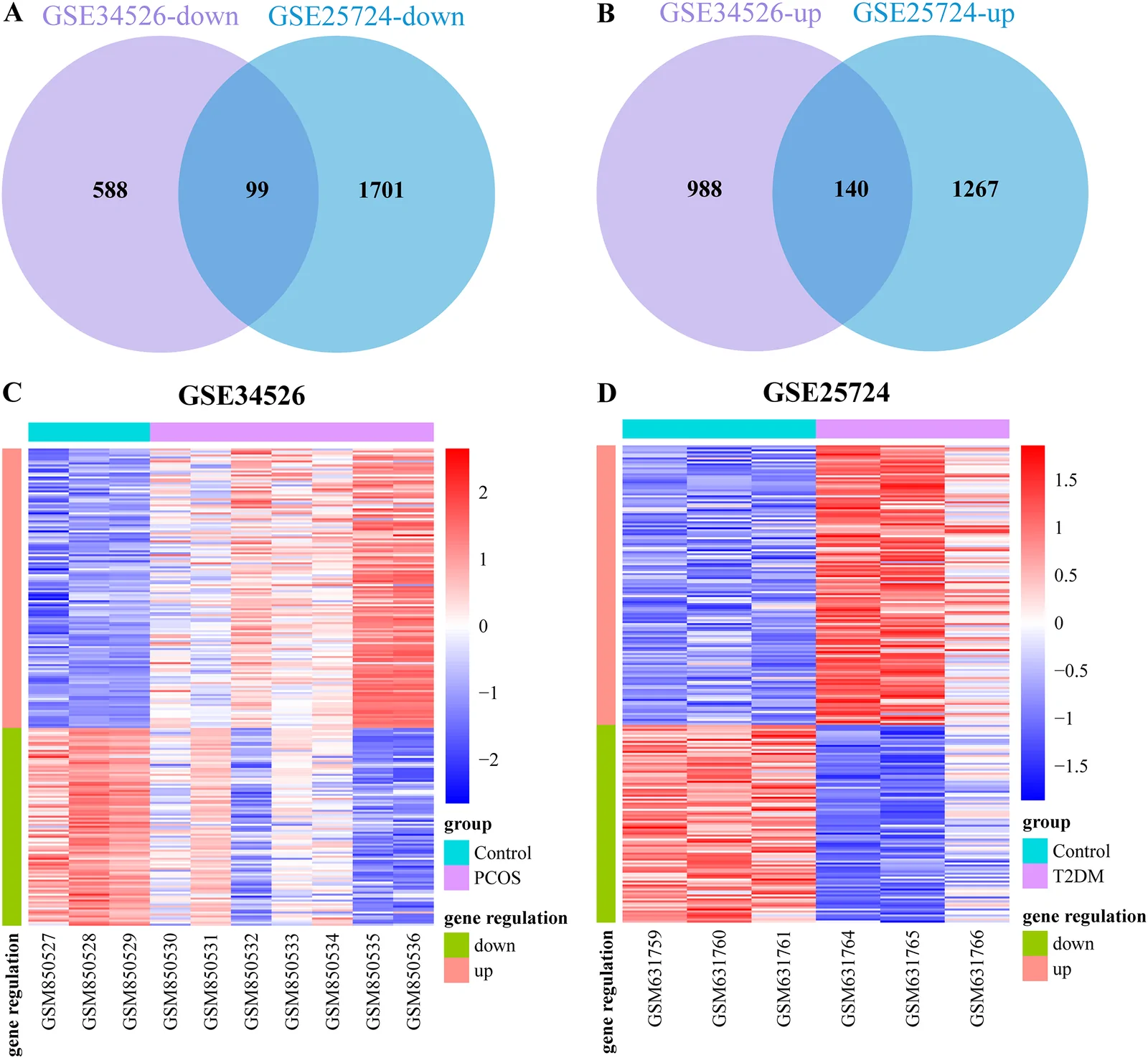
Venn diagrams and heatmaps of common differentially expressed genes (DEGs). **(A)** The Venn diagram of downregulated DEGs in these two datasets; **(B)** The Venn diagram of upregulated DEGs in these two datasets; **(C, D)** The heatmaps of common DEGs in each dataset. Red represents upregulated DEGs, and blue represents downregulated DEGs.

We obtained the GSE34526 dataset for PCOS and GSE25724 dataset for T2DM. The data were neatly distributed in the boxplot after background adjustment and normalization using the RMA method, as illustrated in **Figure 2A, B**. Furthermore, principal component analysis (PCA) revealed that the patient and control groups were well separated (**Figures 2C, D**). Based on the boxplot and PCA results, the processed data were considered reliable and could be further analyzed. Subsequently, as per the rules of |log_2_ FC| > 0.5 and an adjusted *p*-value < 0.05, we identified 1,815 DEGs (1,128 upregulated and 687 downregulated) in the PCOS dataset and 3,207 DEGs (1,407 upregulated and 1,800 downregulated) in the T2DM dataset, respectively. Volcano plots of DEGs in these two datasets are depicted in **Figures 3A, B**. Heat maps of the DEGs are illustrated in **Figure 3C** and D. Venn diagrams were used to intersect these two datasets and obtain 239 common DEGs (140 upregulated and 99 downregulated genes), as illustrated in **Figures 4A, B**. Heat maps of common DEGs in these two datasets are displayed in **Figures 4C, D**.

### 3.2 GO and KEGG pathway enrichment analyses of common DEGs

**Figure 5:**
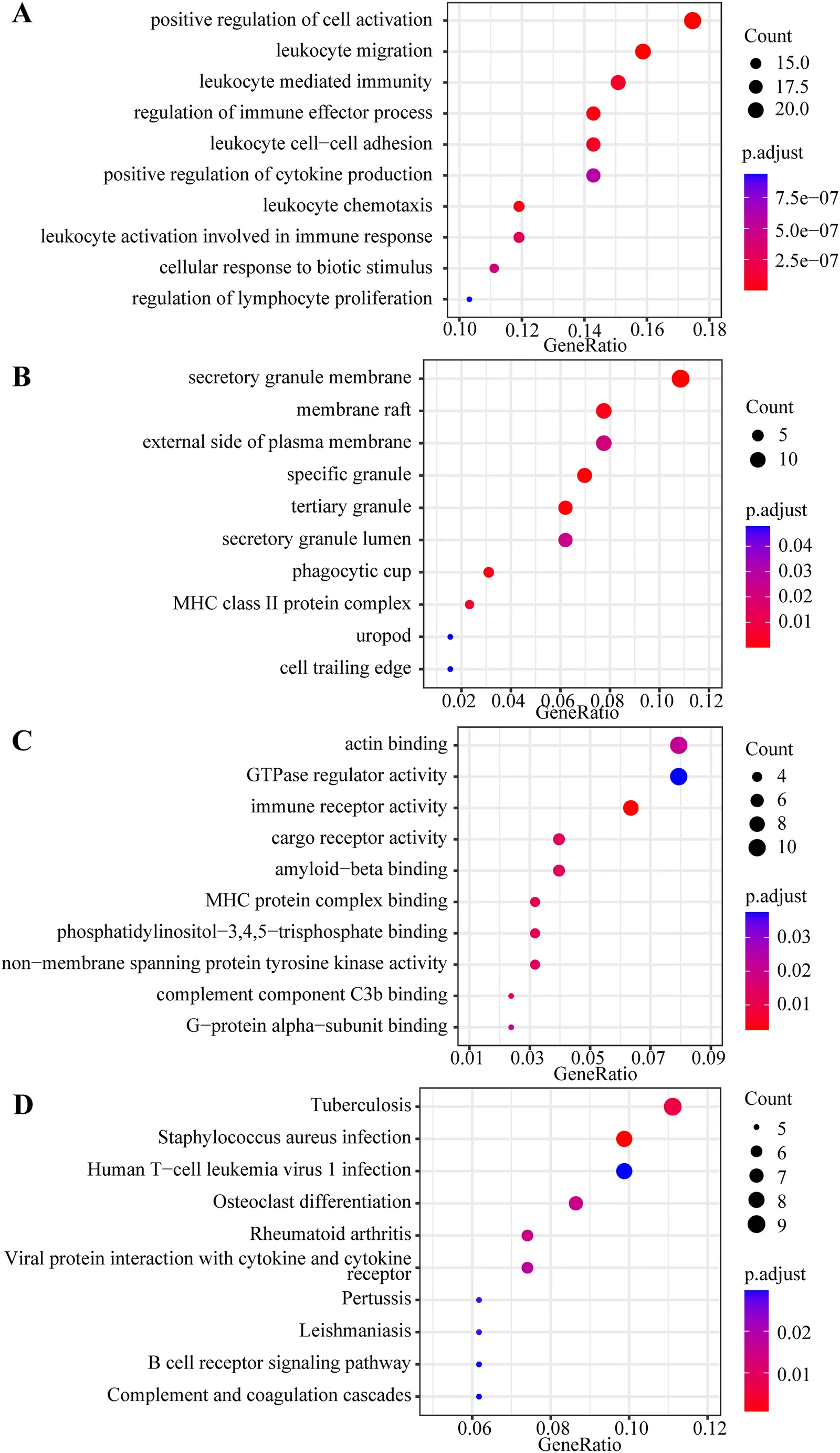
Gene ontology (GO) and kyoto encyclopedia of genes and genome (KEGG) analyses bubble maps of 140 upregulated differentially expressed genes (DEGs). **(A)** The top 10 enriched biological processes (BP) terms of GO analysis; **(B)** The top 10 enriched cellular components (CC) terms of GO analysis; **(C)** The top 10 enriched molecular functions (MF) terms of GO analysis; **(D)** The top 10 enriched KEGG terms. An adjusted *p*-value < 0.05 was considered significant. The x-axis represents the gene ratio of each term. The bubble size represents the count of genes associated with each term: a larger bubble indicates a term with more enriched genes. The bubble color represents the adjusted *p*-value: the redder the color, the lower the adjusted *p*-value.

**Figure 6:**
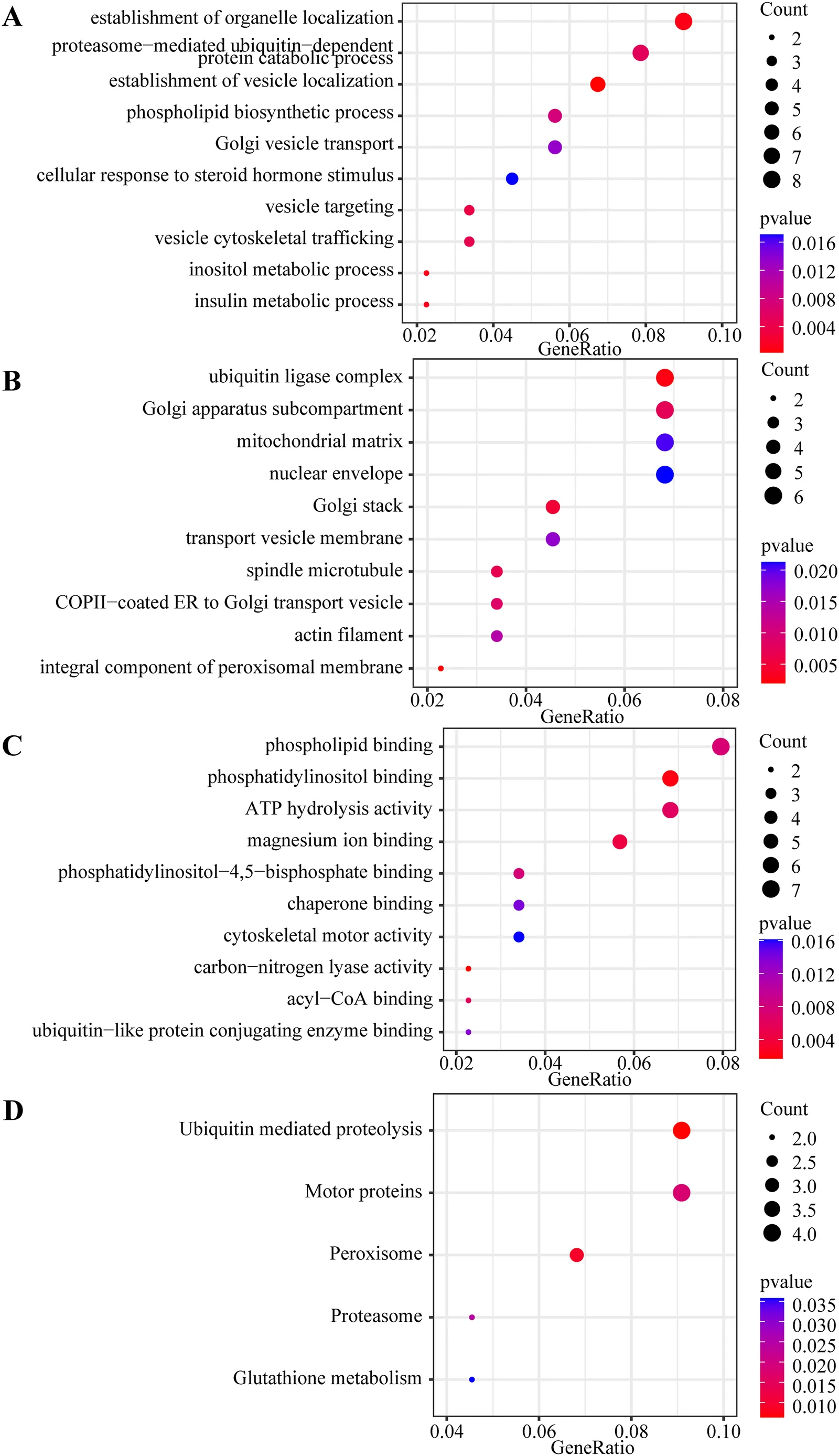
Gene ontology (GO) and kyoto encyclopedia of genes and genome (KEGG) analyses bubble maps of 99 downregulated differentially expressed genes (DEGs). **(A)** The top 10 enriched biological processes (BP) terms of GO analysis; **(B)** The top 10 enriched cellular components (CC) terms of GO analysis; **(C)** The top 10 enriched molecular functions (MF) terms of GO analysis; **(D)** The top 5 enriched KEGG terms. A *p*-value < 0.05 was considered significant. The x-axis represents the gene ratio of each term. The bubble size represents the count of genes associated with each term: a larger bubble indicates a term with more enriched genes. The bubble color represents the *p*-value: the redder the color, the lower the *p*-value.

We conducted GO and KEGG pathway enrichment analyses to investigate the functions of 239 common DEGs. Based on an adjusted *p*-value < 0.05, the top 10 enriched GO and KEGG terms of interest for the 140 upregulated DEGs were identified. As illustrated in **Figures 5A–D**, DEGs were principally enriched in BP, including positive regulation of cell activation, leukocyte migration, leukocyte-mediated immunity, regulation of immune effector processes, leukocyte cell-cell adhesion, positive regulation of cytokine production, leukocyte chemotaxis, and leukocyte activation involved in immune response. Regarding CC, DEGs were primarily enriched in response to secretory granule membrane, membrane raft, external side of the plasma membrane, and specific granules. The MF analysis revealed that DEGs were principally associated with actin binding, GTPase regulator activity, immune receptor activity, cargo receptor activity, amyloid-β binding, and complement component C3b binding. For KEGG enrichment pathways, DEGs were principally enriched in tuberculosis, *Staphylococcus aureus* infection, B cell receptor signaling pathway, and complement and coagulation cascades. Additionally, for the 99 downregulated DEGs, we selected the top 10 enriched GO terms and 5 KEGG terms with *p*-value < 0.05 (**Figures 6A–D**). The enriched KEGG pathways mainly included ubiquitin-mediated proteolysis, motor proteins, and peroxisomes. GO analysis indicated that the downregulated DEGs were predominantly enriched in the establishment of organelle localization, proteasome-mediated ubiquitin-dependent protein catabolic processes, the establishment of vesicle localization in BP, ubiquitin ligase complex, Golgi apparatus subcompartment, Golgi stack in CC, phospholipid binding, phosphatidylinositol binding, and ATP hydrolysis activity in MF. A detailed description of GO and KEGG pathway enrichment analyses is presented in **Supplementary Table 1**.

### 3.3 PPI network construction and modules selection

**Figure 7:**
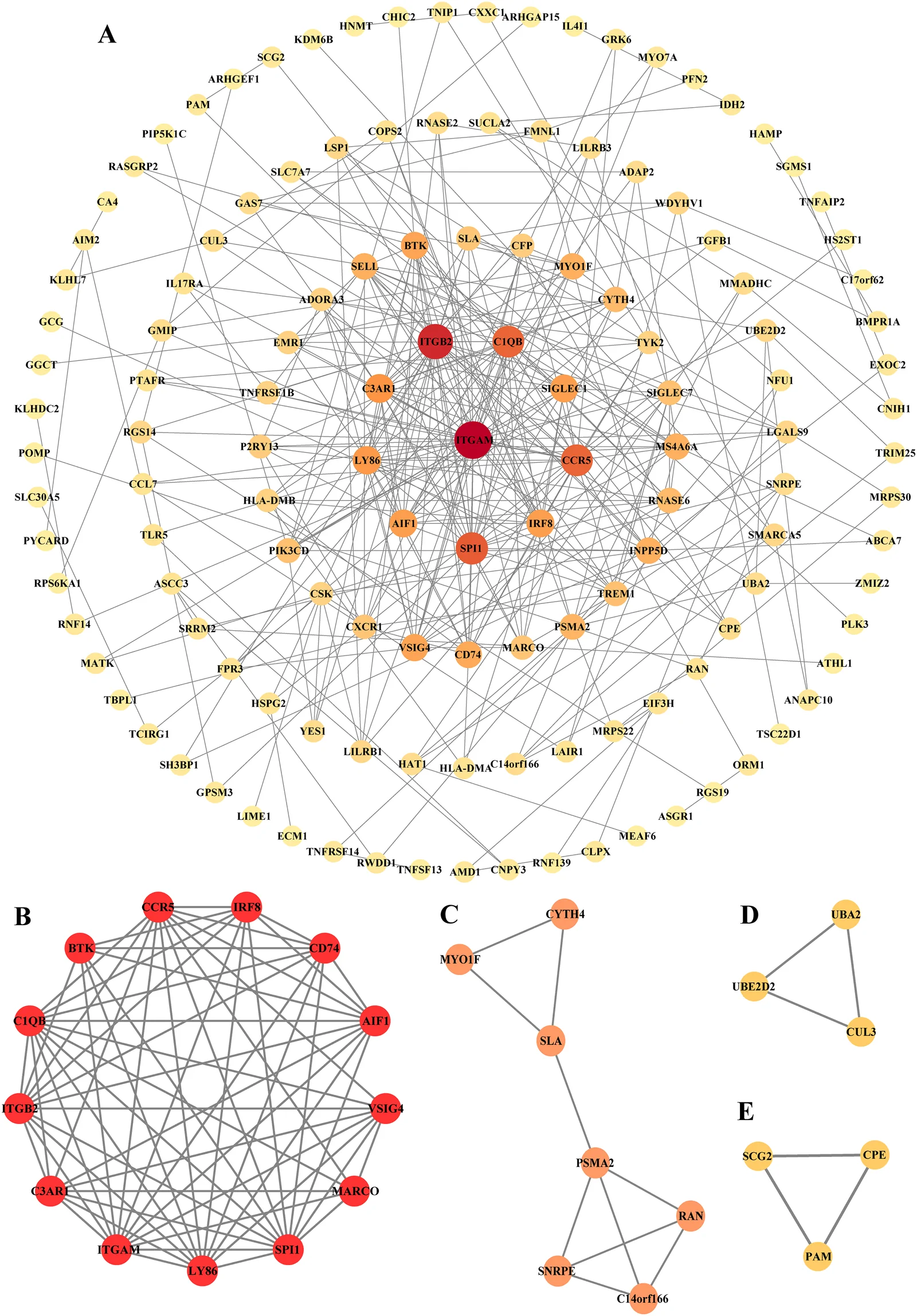
Protein-protein interaction (PPI) network and key modules. **(A)** A PPI network containing 134 nodes and 334 edges. Nodes stand for proteins, and edges correspond to the interactions between two proteins. The size and color of nodes are related to degree scores. A node with a larger size and a darker color has a higher degree score; **(B)** Key module 1 including 13 nodes and 63 edges; the redder the color, the higher the Molecular Complex Detection (MCODE) score. **(C)** Key module 2 including 7 nodes and 10 edges; **(D)** Key module 3 including 3 nodes and 3 edges; **(E)** Key module 4 including 3 nodes and 3 edges. The redder the color,the higher the Molecular Complex Detection (MCODE) score.

To explore the PPI network, 239 common DEGs were entered into the STRING database. The downloaded results with an interaction score > 0.4 and PPI enrichment *p*-value < 10e-16 were imported into Cytoscape to visualize the PPI network. Finally, a PPI network including 134 nodes and 334 edges was constructed (**Figure 7A**). The size and color of the nodes were associated with the degree scores. A node with a larger size and darker color had a higher degree score. Four key modules were identified based on the application of the MCODE plug-in. As shown in **Figure 7B-E** and **Table 4**, cluster 1 had an MCODE score of 10.5, comprising 13 nodes and 63 edges; cluster 2 had an MCODE score of 3.33, comprising 7 nodes and 10 edges; clusters 3 and 4 had an MCODE score of 3, comprising 3 nodes and 3 edges, respectively. Genes in cluster 1, possessing the highest MCODE score, were considered candidate hub genes.

**Table 4:** Scores, nodes and edges of key models.

Cluster	Score (Density*#Nodes)	Nodes	Edges	Node IDs
1	10.5	13	63	VSIG4, AIF1, ITGAM, LY86, C3AR1, ITGB2, BTK, C1QB, CCR5, IRF8, SPI1, MARCO, CD74
2	3.33	7	10	SLA, MYO1F, CYTH4, SNRPE, RAN, PSMA2, C14orf166
3	3	3	3	UBA2, CUL3, UBE2D2
4	3	3	3	PAM, SCG2, CPE

### 3.4 Identification and enrichment analyses of hub genes

**Figure 8:**
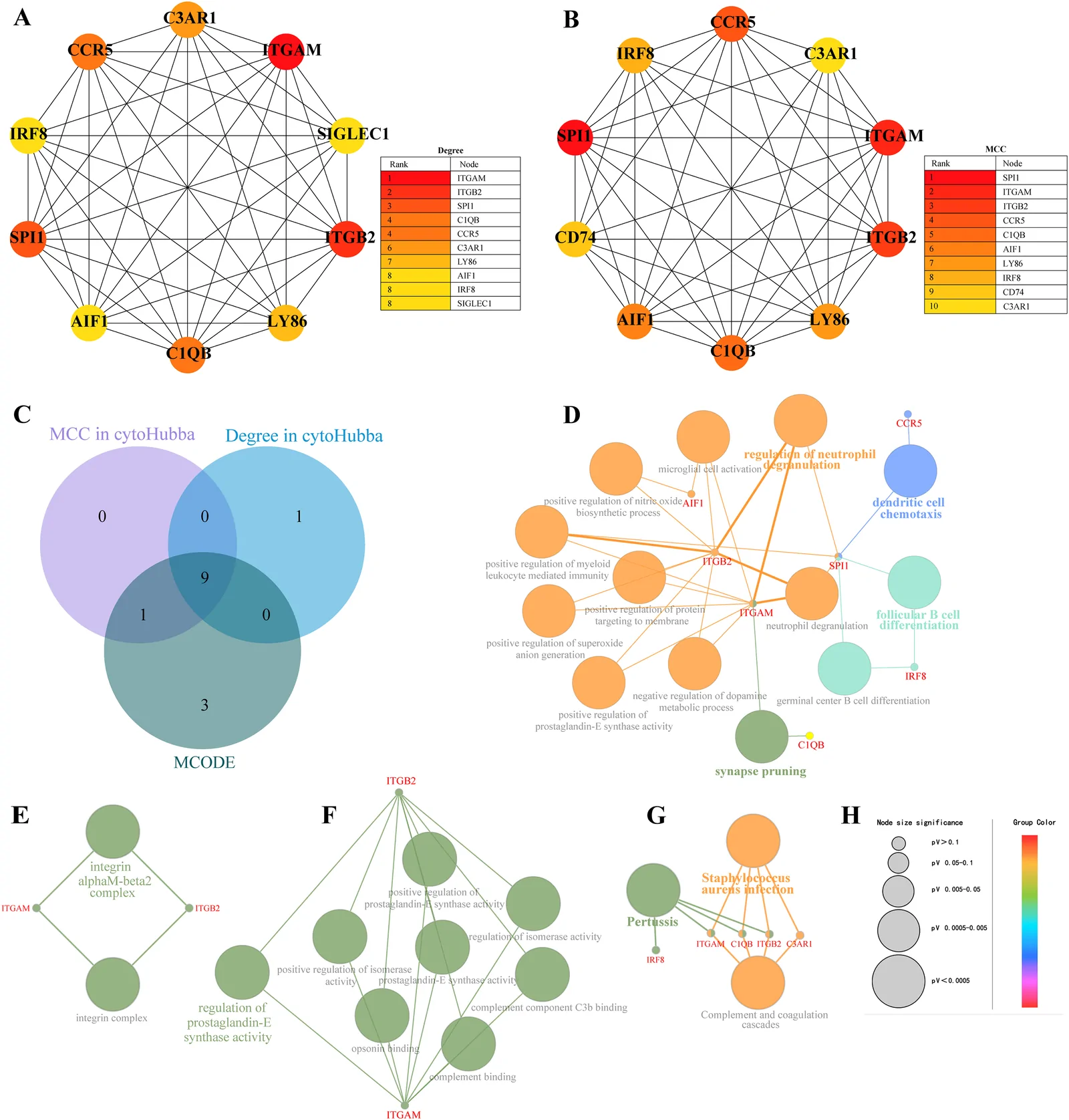
Identification and enrichment analyses of hub genes. **(A)** The top 10 ranked gene networks based on degree scores in the cytoHubba plug-in; **(B)** The top 10 ranked gene networks based on the Maximal Clique Centrality (MCC) algorithm in the cytoHubba plug-in; **(C)** The Venn diagram of hub genes analyzed by the cytoHubba and MCODE plug-in; **(D–G)** The interaction networks of nine hub genes and related biological processes (BP), cellular components (CC), molecular functions (MF) and KEGG pathways constructed and visualized by the ClueGO plug-in; **(H)** The node size represents the *p*-value: the larger the node, the lower the *p*-value.

**Figure 9:**
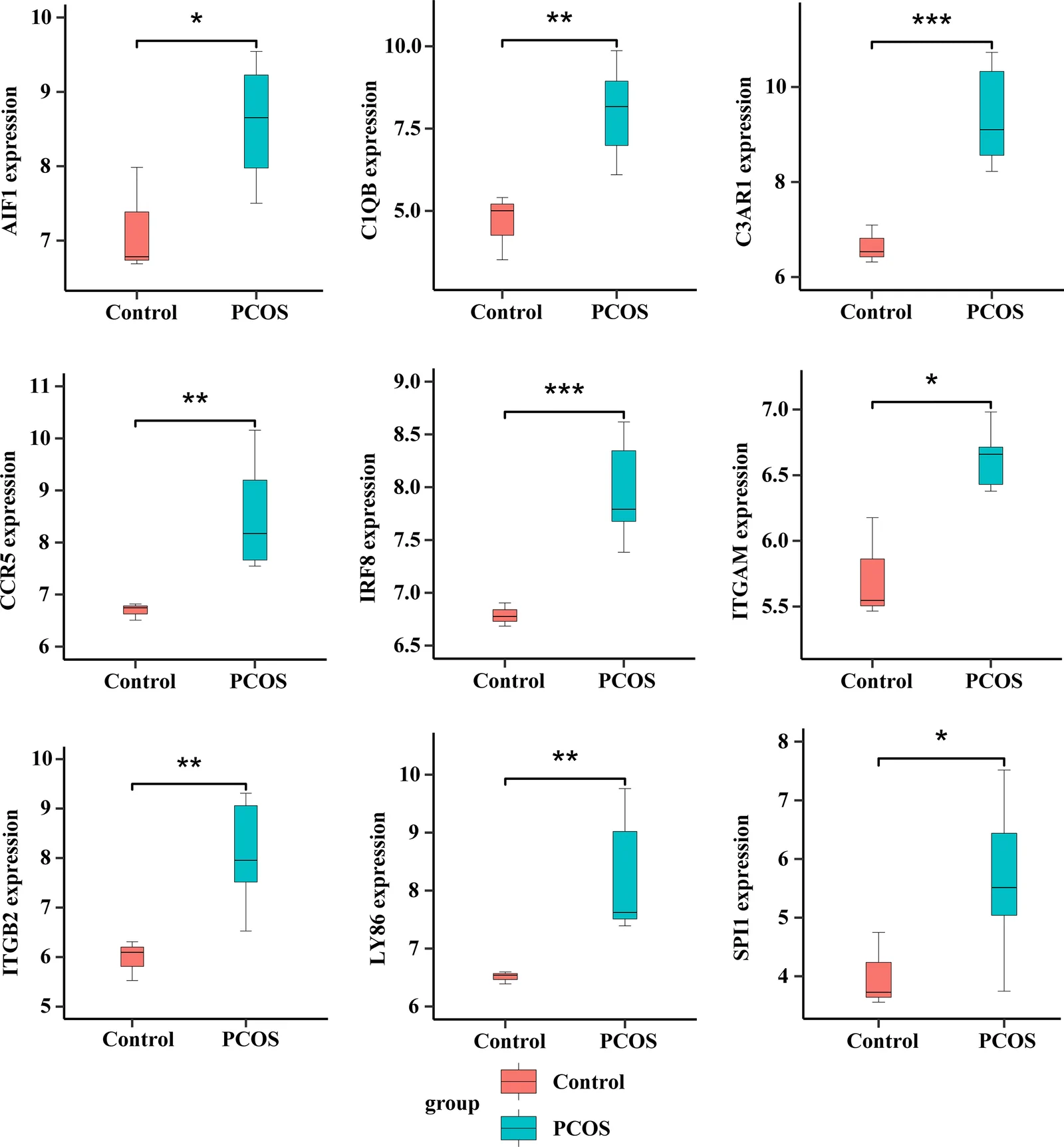
Expression values of nine hub genes in patients with PCOS and normal controls. *p < 0.05; **p < 0.01; ***p < 0.001.

**Figure 10:**
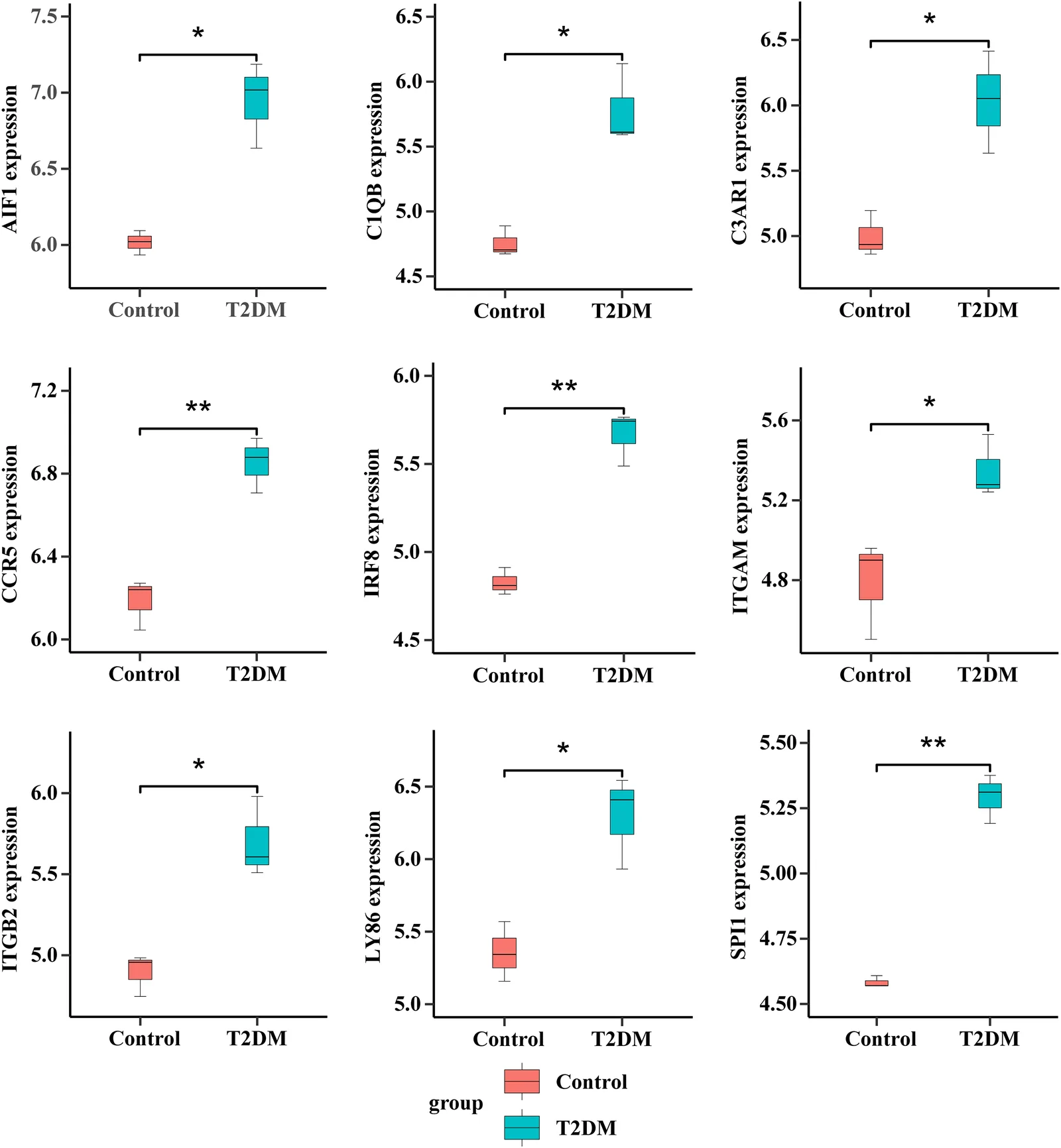
Expression values of nine hub genes in patients with T2DM and normal controls. *p < 0.05; **p < 0.01.

We generated the top 10 ranked genes networks based on the MCC algorithm and degree score, as depicted in **Figure 8A, B**. We used a Venn diagram to intersect genes in cluster 1 and the top 10 ranked genes analyzed by cytoHubba to obtain nine hub genes: ITGAM, ITGB2, SPI1, C1QB, CCR5, C3AR1, LY86, AIF1, and IRF8 (**Figure 8C** and **Table 5**). Detailed information on hub genes is provided in **Supplementary Table 2**. Enrichment analyses revealed a significant association between nine hub genes and the regulation of neutrophil degranulation (ITGAM, ITGB2, and SPI1), dendritic cell chemotaxis (CCR5 and SPI1), follicular B cell differentiation (SPI1 and IRF8), and synapse pruning (ITGAM and C1QB) in BP analysis (**Figure 8D** and **Supplementary Table 3**). The CC and MF analyses revealed that ITGAM and ITGB2 were involved in integrin αM-β2 complex and regulation of prostaglandin-E synthase activity (**Figure 8E, F** and **Supplementary Table 3**). The enriched KEGG pathways primarily included *Staphylococcus aureus* infection (ITGAM, ITGB2, C1QB, and C3AR1) and pertussis (IRF8) (**Figure 8G** and **Supplementary Table 3**). The hub gene expression levels in patients and controls are illustrated in **Figure 9** and **10**, respectively.

**Table 5:** Hub genes ranked in cytoHubba and MCODE plugin of Cytoscape.

Method	MCC in cytoHubba plugin (MCC score)	Degree in cytoHubba plugin (degree score)	MCODE plugin
gene symbol	SPI1 (78101)	ITGAM (35)	VSIG4
	ITGAM 77681)	ITGB2 (30)	AIF1
	ITGB2 (72714)	SPI1 (24)	ITGAM
	CCR5 (72328)	C1QB (23)	LY86
	C1QB (71486)	CCR5 (23)	C3AR1
	AIF1 (60816)	C3AR1 (17)	ITGB2
	LY86 (56796)	LY86 (16)	BTK
	IRF8 (51273)	AIF1 (15)	C1QB
	CD74 (45420)	IRF8 (15)	CCR5
	C3AR1 (26485)	SIGLEC1 (15)	IRF8
			SPI1
			MARCO
			CD74

The common hub genes were bolded based on the above three ranked methods. MCC, Maximal clique centrality; Degree, Node connect degree; MCODE, Molecular complex detection.

### 3.5 Experimental validation of hub genes by RT-qPCR

**Figure 11:**
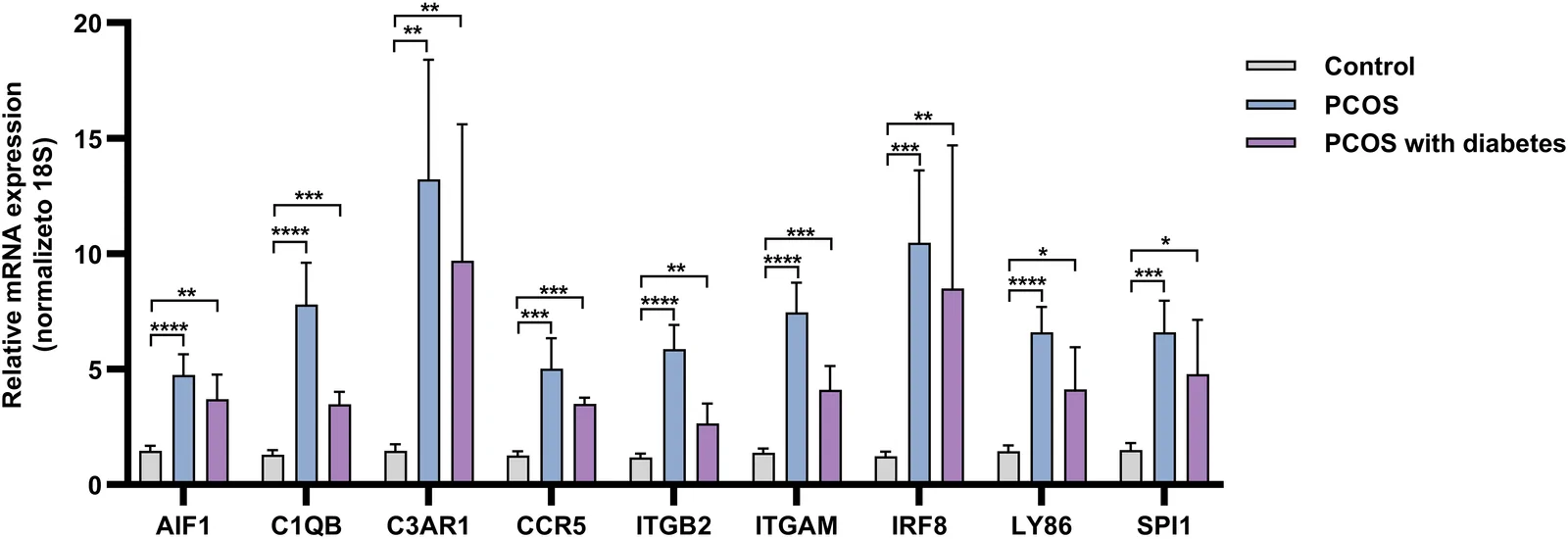
The results of RT-qPCR. *p<0.05, **p<0.01, ***p<0.001, ****p<0.0001.

To enhance the reliability of our findings, we quantified the expression levels of hub genes in the different groups by RT-qPCR (**Figure 11**). Compared with the control group, the mRNA expression levels of ITGAM, ITGB2, SPI1, C1QB, CCR5, C3AR1, LY86, AIF1, and IRF8 are elevated in the PCOS group. Although this upregulation was less pronounced in PCOS with T2DM patients, the differences remained statistically significant. These findings collectively indicate the activation of immuno-inflammatory responses in PCOS patients, regardless of T2DM.

### 3.6 Construction of TF-hub gene network and miRNA-hub gene network

**Figure 12:**
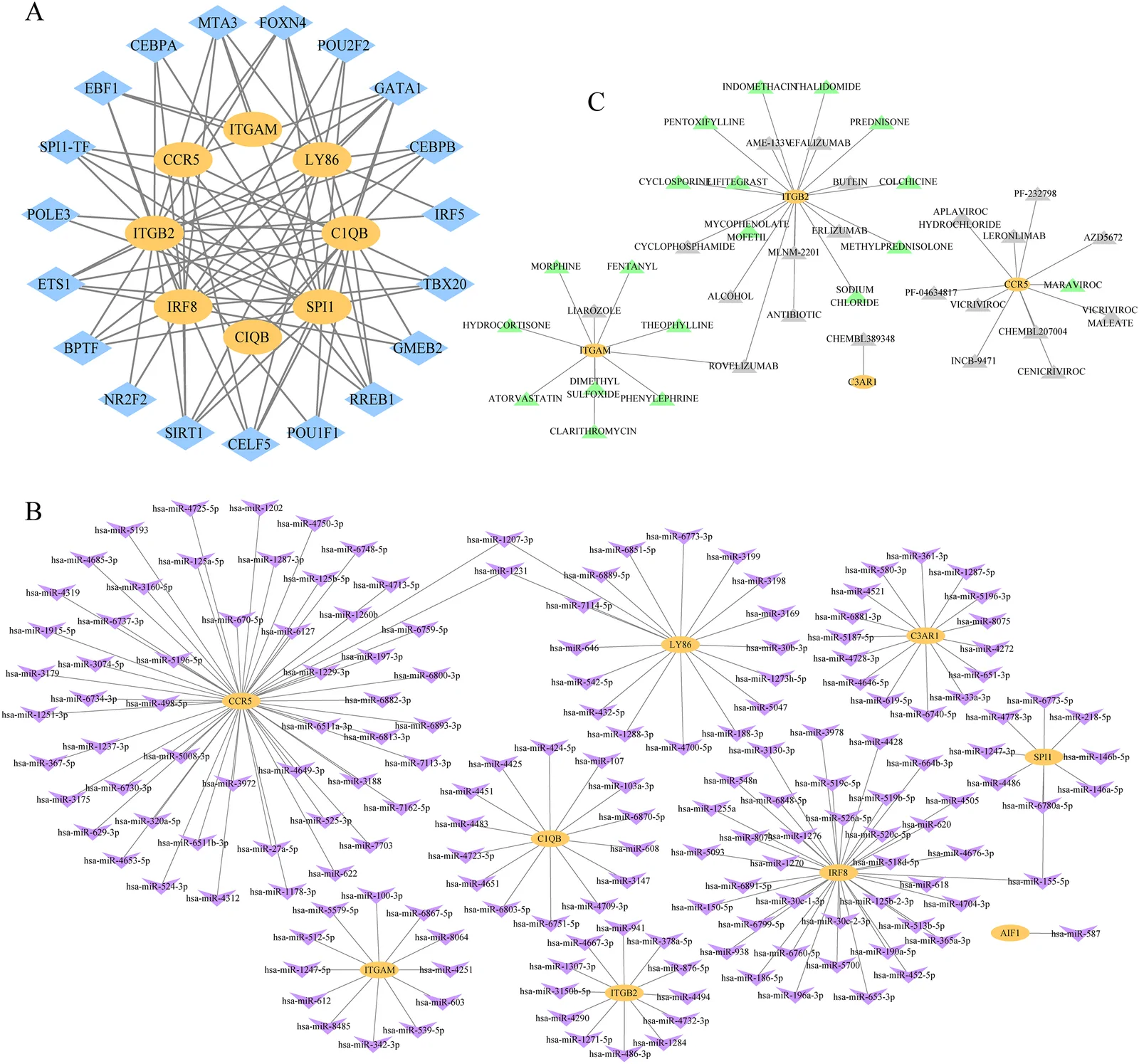
Interaction networks of transcription factor (TF)-hub gene, miRNA-hub gene, and drug-hub gene. **(A)** The TF-hub gene interaction network involved 19 TFs, 8 hub genes, and 93 regulatory pathways; **(B)** The miRNA-hub gene interaction network involved 170 miRNAs, 9 hub genes, and 173 regulatory pathways; **(C)** The drug-hub gene interaction network involved 40 drugs, 4 hub genes, and 41 regulatory pathways. Drugs approved by Food and Drug Administration (FDA) are represented as green triangles, while those not approved are represented as gray triangles. A total of 40 drugs are regularly distributed based on their score levels: the triangle closer to the hub gene has a higher score. TFs are represented as blue diamonds, miRNAs are represented as purple V, and hub genes are represented as yellow ellipses.

We predicted hub gene-related TFs and miRNAs to explore the regulatory molecules and mechanisms in PCOS and T2DM. The iRegulon plug-in was employed to predict the upstream TFs, and a TF-hub gene interaction network involving 19 TFs (FOXN4, ETS1, CELF5, TBX20, GATA1, EBF1, POLE3, BPTF, POU1F1, RREB1, CEBPB, MTA3, SPI1, NR2F2, SIRT1, GMEB2, IRF5, POU2F2, and CEBPA) and 93 regulatory pathways was constructed and visualized (**Figure 12A**). Upon closer examination of the network diagram, we observed that multiple TFs could regulate a hub gene, and a TF could regulate multiple target genes. Moreover, SPI1 functions as a TF. The miRNAs that may regulate hub genes were also predicted through four databases, and a miRNA-hub gene network regulation diagram composed of 179 nodes and 173 edges was created using Cytoscape software (**Figure 12B**). Three miRNAs interacted with multiple genes: hsa-miR-155-5p targeting SPI1 and IRF8, has-miR-1231 targeting CCR5 and LY86, and hsa-miR-1207-3p targeting CCR5 and LY86.

### 3.7 Drug-gene interaction analysis

We entered hub genes into the DGIdb database to predict potential drugs in patients with PCOS and T2DM. Forty drugs capable of targeting hub genes were selected to construct a visual network (**Figure 12C**). Drugs are represented by triangles and regularly distributed based on their interaction scores. A triangle closer to the center has a higher interaction score, indicating greater credibility. Drugs approved by the Food and Drug Administration (FDA) were displayed in green, whereas those not approved were displayed in gray. Most of these drugs are inhibitors. ROVELIZUMAB is a humanized anti-CD11/CD18 monoclonal antibody targeting ITGAM and ITGB2. We listed detailed drug information in **Supplementary Table 4**, including interaction type, sources, PMIDs, query scores, interaction scores, and FDA approval status.

## 4 Discussion

PCOS and T2DM are chronic endocrine and metabolic diseases associated with multiple genes and pathogenesis (3, 46). The bidirectional relationship between these two conditions has been well documented. However, the shared molecular mechanisms remain incompletely elucidated. The vicious cycle of androgen excess and IR represents a vital pathogenic link, closely associated with chronic low-grade inflammation and immune system dysfunction (47, 48). However, the specific genetic factors mediating this interconnection have not been systematically explored. In this study, we aimed to explore shared immune- and inflammation-related genes and pathways in PCOS with T2DM using bioinformatics analysis, followed by experimental validation and drug prediction.

Through integrated bioinformatics analysis of two independent microarray datasets, we identified 239 common DEGs, with functional enrichment analyses revealing strong associations with immune regulation and inflammatory responses. Subsequent PPI network analysis and module selection led to the identification of nine hub genes: ITGAM, ITGB2, SPI1, C1QB, CCR5, C3AR1, LY86, AIF1, and IRF8. All nine genes were significantly upregulated in both PCOS and T2DM datasets, a finding that was further validated by RT-qPCR in independent clinical samples. These results point to a shared immuno-inflammatory signature underlying both conditions.

Among the identified hub genes, ITGAM and ITGB2, encoding the integrin αM and β2 chains respectively, together form the Mac-1 (CD11b/CD18) complex (49). Mac-1 is highly expressed in dendritic cells, neutrophils, monocytes and macrophages and is involved in cell activation, degranulation, chemotaxis, migration, adhesion, and phagocytosis (50, 51). Our finding that both genes are upregulated in PCOS and T2DM datasets aligns with previous reports of elevated CD11b/CD18 complex expression in T2DM patients and increased CD11b expression in PCOS rodent models (52–55). Mechanistically, Mac-1 functions as a pro-inflammatory molecule that interacts with multiple ligands, including intercellular adhesion molecule-1 (ICAM-1), complement protein iC3b, and fibrinogen, thereby promoting vascular inflammation and tissue damage (56–58). These interactions may contribute to the increased metabolic risk observed in women with PCOS and T2DM (11, 59). Notably, our enrichment analyses further revealed that ITGAM and ITGB2 are involved in neutrophil degranulation and the regulation of prostaglandin-E synthase activity, suggesting a broader role in modulating inflammatory mediator production.

The complement system emerged as another key pathway through our identification of C1QB and C3AR1 as hub genes. C3AR1 encodes the receptor for C3a, a fragment cleaved by C3 (60), while C1QB encodes the C1q B-chain, a crucial component of the classical complement pathway (61). Our findings extend previous observations that complement activation is implicated in both T2DM and PCOS. Elevated C3 and C3a levels have been associated with IR and increased T2DM risk in prospective cohort studies (62), and C3aR knockdown has been shown to improve IR and adipose tissue inflammation in high-fat diet (HFD)-induced mice (63). Additionally, C1QB expression was elevated in the kidneys of patients with diabetic kidney disease (DKD) and was inversely correlated with the glomerular filtration rate (GFR) (64). C1QB also contributes to pancreatic β-cell destruction by promoting macrophage proliferation and differentiation, thereby disrupting the immune microenvironment (65). A cross-sectional study from China showed that women with PCOS had higher C3 levels than BMI-matched non-PCOS women, with an inverse relationship between C3 and insulin sensitivity (66). Notably, women with insulin-resistant PCOS had higher plasma C3 and C3a levels than those with insulin-sensitive PCOS, suggesting that IR may exacerbate complement dysfunction (67). Our identification of C1QB and C3AR1 as shared upregulated genes provides a direct genetic link supporting the involvement of the complement cascade in the comorbidity of PCOS and T2DM.

SPI1 (PU.1) and IRF8 are key transcription factors involved in immune cell development and inflammatory responses (68, 69). Our finding that SPI1 is upregulated aligns with recent evidence that PU.1 knockdown improves glucose metabolism and hepatic inflammation in diabetic db/db mice (70). It is also consistent with the report that SPI1 overexpression increases NR4A1 transcriptional activity, which is closely associated with the pathophysiology of PCOS (71). Meanwhile, IRF8 has been implicated in diabetic complications. Advanced glycation end products (AGEs) induce macrophage autophagy and M2-to-M1 polarization through IRF8 activation, impairing diabetic wound healing (72). Moreover, another study revealed that spleen tyrosine kinase deficiency (SYK) improved retinal microglial inflammation in diabetic mice by inhibiting IRF8 and PU.1 expression, thus alleviating diabetic retinopathy (73).

The chemokine receptor CCR5 plays a vital role in leukocyte activation and migration, and pro-inflammatory cytokines production (74). CCR5 can induce IR and chronic systemic inflammation to facilitate T2DM (75). Animal studies have demonstrated that CCR5 inhibitors improve obesity-induced IR, pancreatic β-cell dysfunction, and chronic systemic inflammation by suppressing macrophage infiltration and polarization in the adipose and pancreatic tissue (76, 77). Multiple clinical studies have indicated that CCR5 gene polymorphisms are closely related to the T2DM phenotype and an increased risk of T2DM nephropathy (78–80). In PCOS, elevated plasma CCL5 levels and CCR5 overexpression in adipose tissue correlate positively with IR and testosterone levels (81). LY86 (MD-1) and AIF1 function as immuno-inflammatory modulators (82, 83). MD-1 deficiency alleviated IR and adipose tissue inflammation in HFD-induced mice (82), while AIF1 may accelerate diabetes development by inducing islet inflammation and impairing insulin secretion (84, 85). Collectively, these findings position the nine hub genes as central nodes in a shared immuno-inflammatory network linking PCOS and T2DM.

PCOS and T2DM are both recognized as chronic low-grade inflammatory diseases, characterized by elevated circulating pro-inflammatory cytokines and immune dysregulation (86, 87). Our identification of shared immune- and inflammation-related hub genes provides a molecular basis for this clinical overlap. In PCOS, hyperandrogenism stimulates neutrophil activity and M1 macrophage polarization while inhibiting T and B lymphocytes activity, eosinophil activity, and dendritic cell function, thereby resulting in an imbalance of cytokines produced by immune cells and chronic low-grade inflammation (88). This inflammatory milieu, in turn, can further promote androgen production (89). Concurrently, excess androgen facilitates the deposition of abdominal and visceral adipose tissue, which is a key driver of IR and compensatory hyperinsulinemia (1). Moreover, chronic low-grade inflammation can also directly induce IR (90). IR not only accelerates the progression to T2DM, but also perpetuates a state of chronic inflammation through the activation of multiple inflammatory signaling pathways (86). Furthermore, hyperinsulinemia can directly stimulate excessive androgen secretion by increasing 17, 20-lyase activity in the adrenal cortex and ovarian theca cells (3, 91). It can also enhance the amplitude and frequency of luteinizing hormone (LH) secretion and increase free testosterone concentration by reducing the production of sex hormone-binding globulin (91, 92). In addition, IR promotes adipose androgen synthesis by upregulating aldoketoreductase type 3 activity in the subcutaneous adipose tissue of women with PCOS (93). Thus, hyperandrogenism interacts with IR, forming a vicious cycle involving inflammatory and immune responses. However, this pathophysiological loop does not occur in all individuals with PCOS. It is important to distinguish cases in which PCOS develop independently of T2DM from those in which PCOS and T2DM are pathophysiologically interconnected. In a subset of individuals, particularly those with normoandrogenic or lean PCOS phenotypes, the condition may arise primarily from ovarian dysfunction or neuroendocrine abnormalities, with minimal contribution from metabolic dysfunction. In these cases, the risk of progressing to T2DM may be relatively lower. Conversely, in individuals with hyperandrogenic PCOS phenotype, especially when accompanied by obesity, IR serves as a core pathogenic bridge, increasing the susceptibility to T2DM (94). The hub genes identified in our study may represent the molecular basis of this metabolically driven subtype. Further research is necessary to explore whether the expression of these genes can serve as biomarkers to stratify PCOS patients based on their risk of metabolic complications, ultimately guiding more personalized and effective therapeutic strategies.

To further explore the regulatory landscape, we constructed miRNA-hub and TF-hub gene networks (95, 96). Among the predicted miRNAs, miR-155-5p targeting SPI1 and IRF8 has been shown to enhance glucose homeostasis and insulin sensitivity (97, 98)., and improve follicular development in PCOS (99), suggesting a potential therapeutic role. Moreover, earlier studies have verified that miR-155-5p negatively regulates SPI1 expression (100). Therefore, we supposed that miR-155-5p can exert a beneficial effect on PCOS and T2DM by downregulating SPI1 expression. A recent study indicated that miR-1231 is associated with inflammation-related pathways (101). We speculated that miR-1231 may exert functions in the pathogenesis of PCOS and T2DM by regulating inflammatory responses…. Several TFs identified, including CEBPB, CEBPA, SPI1, ETS1, EBF1, GATA1, and SIRT1 have established roles in inflammation, insulin sensitivity, and reproductive function. Notably, SPI1 itself functions as a TF, and its interaction with ETS1 may represent a key regulatory axis in PCOS pathogenesis (70, 102–107)…… Drug-gene interaction analysis identified 40 potential therapeutic drugs targeting hub genes. Of particular interest, Maraviroc, Cenicriviroc, and PF-04634817 targeting CCR5 have demonstrated efficacy in ameliorating IR, pancreatic β-cell dysfunction, and inflammation in preclinical models (77, 108, 109). Butein targeting ITGB2 and DMSO targeting ITGAM exhibit anti-inflammatory and insulin-sensitizing properties (110, 111)…. Rovelizumab, a humanized anti-CD11/CD18 monoclonal antibody, targets both ITGAM and ITGB2 and has been shown to alleviate inflammatory responses (112). These findings provide a foundation for drug development strategies in PCOS and T2DM.

Earlier studies conducted separate investigations on the roles of immunity and inflammation in PCOS and T2DM via bioinformatics analysis (113, 114). However, the shared immuno-inflammatory mechanisms linking these two conditions remain largely unexplored. This study offers novel perspectives on the biological mechanisms associated with inflammation and immunity in patients with PCOS and T2DM. Furthermore, potential miRNAs, TFs, and targeted drugs were identified. However, some limitations exist in this study. First, the data analyzed in the present study were sourced from existing GEO datasets, and the datasets meeting our requirements had limited sample sizes. Therefore, the results may have slight deviations and should be interpreted with caution. Second, this work was primarily reliant on bioinformatics analysis. These hub genes have been validated in different datasets. We also performed RT-qPCR validation using clinical samples. However, the sample size was relatively small (14 healthy controls, 11 patients with PCOS, and 3 patients with PCOS and T2DM), and the age range was narrow (25–38 years). Given that aging may influence chronic low-grade inflammation (115), we were unable to perform age-stratified analysis. Therefore, larger cohorts with broader age distributions are warranted to further validate our findings. Third, in future studies, particularly when selecting specific hub genes for further functional investigation, we will prioritize correlation analyses with clinical and molecular markers to validate which genes are most relevant to the pathogenesis of PCOS and T2DM.

## 5 Conclusion

We identified shared immune- and inflammation-related genes and molecular mechanisms associated with PCOS and T2DM through bioinformatics analysis and experimental validation. Nine hub genes (ITGAM, ITGB2, SPI1, C1QB, CCR5, C3AR1, LY86, AIF1, and IRF8) were significantly upregulated and may play significant roles in the pathogenesis of PCOS and T2DM by regulating immune cell activity and inflammatory responses. Hub genes associated with immune regulation and inflammatory responses were also involved in IR and glucose metabolism. Several miRNAs (miR-155-5p, miR-1231, and miR-1207-3p) and TFs (CEBPB, CEBPA, SPI1, ETS1, EBF1, GATA1, and SIRT1) are associated with IR, inflammatory responses, follicular development, and androgen metabolism. Forty drugs targeting these hub genes were screened through the DGIdb database. Among them, maraviroc, cenicriviroc, PF-04634817 (targeting CCR5), butein (targeting ITGB2), DMSO (targeting ITGAM), and rovelizumab (targeting both ITGB2 and ITGAM) appear to have potential efficacy in treating PCOS with T2DM. This study is the first to give unique insights into the pathogenesis and therapeutic strategies for PCOS with T2DM from the perspectives of immunity and inflammation. However, our results should be confirmed through further experimental and clinical studies.

## Data Availability

The original contributions presented in the study are included in the article/**Supplementary Material**. Further inquiries can be directed to the corresponding authors.
