# Correction: Shared immune-inflammatory gene networks and drug prediction in polycystic ovary syndrome and type 2 diabetes mellitus: a bioinformatics and experimental validation study

**DOI:** 10.3389/fendo.2026.1858399

**Published:** 2026-04-24

**Authors:** Xuemeng Liu, Yanlei Wang, Qiuhan Bi, Ying Zhang, Shumin Wang, Pan Yang, Jie Pei, Weixi Zhu, Yijing Chen, Zhiguo Zhang, Beili Chen, Qiu Zhang, Yi Zhang, Tian Jiang

**Affiliations:** 1 Department of Science and Education, The Second People’s Hospital of Hefei, Hefei Hospital Affiliated to Anhui Medical University, Hefei, Anhui, China; 2 Department of Endocrinology, The First Affiliated Hospital of Anhui Medical University, Hefei, Anhui, China; 3 Department of Obstetrics and Gynecology, The First Affiliated Hospital of Anhui Medical University, Hefei, Anhui, China

**Keywords:** drugs prediction, gene regulatory networks, immunity, inflammation, polycystic ovary syndrome, type 2 diabetes mellitus

Author Xuemeng Liu was erroneously omitted as equal contributing author. The dagger symbol (†), used to indicate equal contribution, was missing next to her name in the author list.

The original version of this article has been updated.

