# Correction: Shared immune-inflammatory gene networks and drug prediction in polycystic ovary syndrome and type 2 diabetes mellitus: a bioinformatics and experimental validation study

**DOI:** 10.3389/fendo.2026.2015080

**Published:** 2026-10-21

**Authors:** Xuemeng Liu, Yanlei Wang, Qiuhan Bi, Ying Zhang, Shumin Wang, Pan Yang, Jie Pei, Weixi Zhu, Yijing Chen, Zhiguo Zhang, Beili Chen, Qiu Zhang, Yi Zhang, Tian Jiang

**Affiliations:** 1 Department of Science and Education, The Second People’s Hospital of Hefei, Hefei Hospital Affiliated to Anhui Medical University, Hefei, Anhui, China; 2 Bengbu Medical University, Bengbu, Anhui, China; 3 Department of Endocrinology, The First Affiliated Hospital of Anhui Medical University, Hefei, Anhui, China; 4 Department of Obstetrics and Gynecology, The First Affiliated Hospital of Anhui Medical University, Hefei, Anhui, China

**Keywords:** drugs prediction, gene regulatory networks, immunity, inflammation, polycystic ovary syndrome, type 2 diabetes mellitus

The funder “Natural Science Key Project of Bengbu Medical University, 2025byzd124” was erroneously omitted.

The original version of this article has been updated.

